# Number of pathologists in Germany: comparison with European countries, USA, and Canada

**DOI:** 10.1007/s00428-020-02894-6

**Published:** 2020-07-27

**Authors:** Bruno Märkl, Laszló Füzesi, Ralf Huss, Svenja Bauer, Tina Schaller

**Affiliations:** General and Special Pathology, Faculty of Medicine, University Augsburg, Augsburg, Germany

**Keywords:** Number of pathologists, Workload, Shortage of staff, Working models

## Abstract

The rapid development of pathology is in contrast to a shortage of qualified staff. The aims of the present study are to compile basic information on the numbers of German physicians in pathology and to compare it with the situation in Europe and overseas. In addition, model calculations will shed light on the effects of part-time working models. Various publicly accessible databases (EuroStat) as well as publications of medical associations and professional associations of European countries and the USA/Canada were examined. In addition, a survey was carried out among the institutes of German universities. Figures from 24 European countries and the USA/Canada were evaluated. With one pathologist per 47,989 inhabitants, the density of pathologists in Germany in relation to the population is the second-lowest in Europe (average: 32,018). Moreover, the proportion of pathologists among the physicians working in Germany is the lowest in Europe and at the same time lower than in the USA and Canada (Germany: 1:200, USA: 1:70, Canada: 1:49). The ratio of pathologists to medical specialists is shifted in the same direction. The survey among university pathologists revealed a relevant increase in the workload over the last 10 years. The majority of institutes can manage this workload only with considerable difficulties. With a ratio between specialists and residents of 1:1, the university institutes show a high commitment in the area of training. The results of this study indicate a shortage of pathologists in Germany that could lead to a bottleneck in large parts of the health system.

For some time now, reports have been accumulating about an increasing shortage of healthcare personnel not only in Germany but also in other parts of Europe and North America. This applies primarily to nursing care but also parts of the medical profession. Here, it is primarily general medicine and anesthesia, but also numerous other disciplines, which are confronted with considerable problems [1–7]. These personnel bottlenecks lead to relevant difficulties in the care of patients with diseases of all degrees of severity, both in the outpatient and inpatient sector. Furthermore, they affect conservative as well as operative medicine [8–12] and present the health care system with considerable challenges.

Because of the overarching effects, bottlenecks are also particularly relevant in smaller cross-sectional disciplines such as pathology, since despite a relatively small lack of forces in absolute terms, a system-critical effect nevertheless occurs.

The fact that staff shortages in pathology are a problem that affects many and possibly all countries is shown by various reports and initiatives by national professional associations and pathologists [13, 14]. In addition, there are also indications that the situation in Germany is particularly serious, due to the workload that is particularly high by international standards in the form of large numbers of cases and a high time burden due to interdisciplinary conferences. The generally increased use of part-time models could lead to an increased shortage of personnel. It is the aim of this study to obtain reliable figures that allow international comparison. A survey of university institutes in Germany will provide information on the development of the past few years and the current situation. In addition, models are to be calculated that show the effects of different working time models. This work touches a political and socially sensitive issue. It is neither the aim of this study to questioning the widely accepted positive aspects of part-time working nor to criticize changes concerning the attitude to a work-life-balance between the generations.

## Materials and methods

The numbers of active physicians and specialist groups in numerous European countries (Austria, Belgium, Bulgaria, Croatia, Estonia, France, Germany, Iceland, Ireland, Italy, Latvia, Lithuania, Malta, the Netherlands, Norway, Poland, Portugal, Romania, Serbia, Slovenia, Spain, Switzerland, United Kingdom, Turkey), the United States, and Canada were obtained from various databases or publicly available information sources of professional associations and independent non-profit organizations: the data of the European countries from 2017 were obtained from the EuroStat database [15]. Swiss data for 2017 were taken from the Swiss doctors’ association (FMH) physician statistics [16]. US data from 2017 were taken from a publication of the *Association of American Medical Colleges* [17] and the Canadian data from 2018 from a publication of the *Canadian Institute for Health Information* [18]. These figures refer to medical specialists.

Four hypothetic exemplary working time models were created comparing uninterrupted work in full-time with corresponding part-time models with work interruptions to illustrate their effects.

In addition, an anonymous online survey was conducted among 37 university institutes of pathology in Germany, which included questions on *current case numbers*, *case number developments*, *personnel requirements for case conferences*, *number of physicians employed and proportion of part-time staff*, *stress development*, *coping with requested services*, *the performance of Generation Y* (*born 1980–1999*).

## Results

### Number of pathologists, rate of pathologists in relation to population, and other groups of doctors

**Fig. 1:**
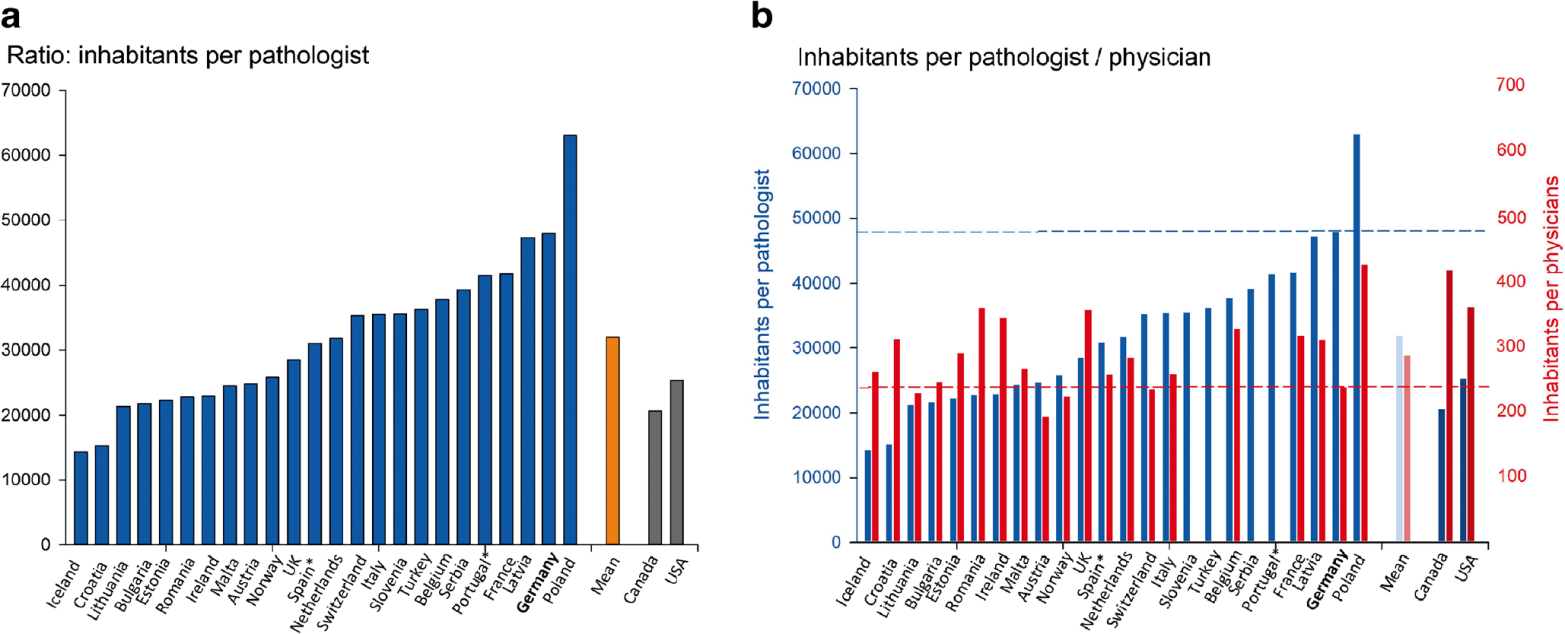
Doctor densities 2017 in European countries. **A** Population per pathologist. Germany has the second lowest density of pathologists per inhabitant after Poland. **B** Ratio of pathologists per inhabitant (blue bars - left *Y*-axis) and general physician density (red bars - right *Y*-axis). Germany has the fourth highest general physician density, which leads to a strong imbalance between the total number of physicians and pathologists. Source: https://appsso.eurostat.ec.europa.eu/nui/show.do?dataset=hlth_rs_specang=en

**Fig. 2:**
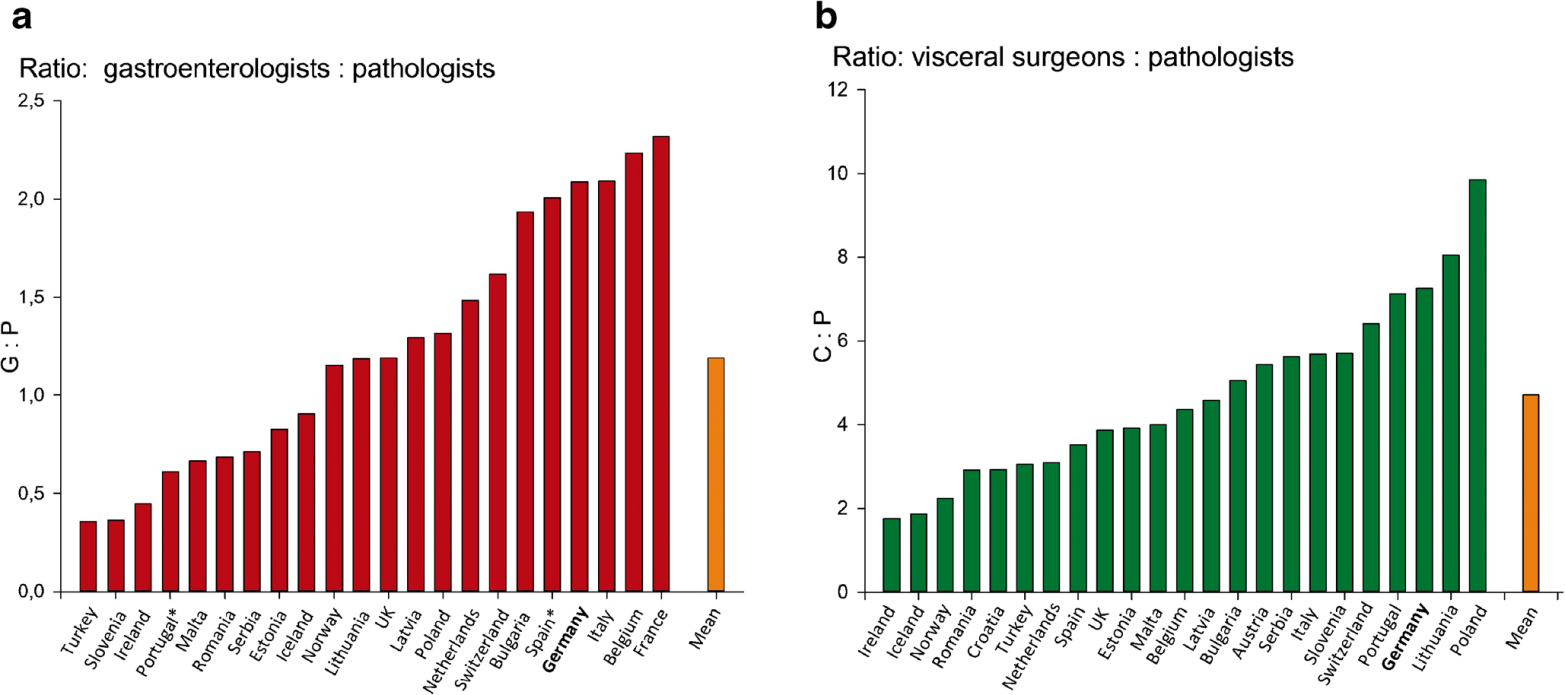
Ratio of sample-generating groups of specialists to pathological consequences 2017. **A** Gastroenterologists to pathologists. In Germany, there is one pathologist for every two gastroenterologists. In half of the countries, the ratio is balanced or shifted in favor of the pathologies. **B** Visceral surgeons to pathologists. In Germany, one pathologist examines the preparations of about seven visceral surgeons, the average is four surgeons per pathologist. Source: https://appsso.eurostat.ec.europa.eu/nui/show.do?dataset=hlth_rs_specang=en

The number of pathologists working within one of the European countries listed above ranges from 18 (Malta) to 2271 (United Kingdom) in 2017. There are 261 pathologists working in Switzerland. In Canada and the USA, the numbers are 1767 and 12,839, respectively. A total of 1692 pathologists are registered in Germany. The number of inhabitants of a country per one pathologist varies in the valuated European countries between 14,309 (Island) and 63,028 (Poland) and is on average 32,018 ± 11,445. In Switzerland, this number is 35,355, in Canada and the USA 20,658 and 25,325, respectively. In Germany, there is one pathologist per 47,989 inhabitants (Fig. 1A). In contrast, the general physician density in Germany, with one physician per 240 inhabitants, is among the highest among the countries studied, with figures ranging from 195 (Austria) to 430 (Poland) in Europe (mean value: 289 ± 56) and 237 in Switzerland. Relatively low general physician densities were found overseas with one physician per 421 inhabitants in Canada and 364 inhabitants in the USA. This results in a pathologist-physician ratio of 1:200, which is the lowest under the analyst countries (mean: 108 ± 39). From the point of view of pathologists working in Germany, a particularly unfavorable situation arises here, since the density of pathologists is among the lowest in international comparison, while the density of physicians is generally particularly high (Fig. 1B). This is also reflected in the numerical ratio between other groups of specialists and pathologists. For example, the ratio between gastroenterologists and visceral surgeons, which are important creators of samples that are evaluated by pathologists, in Germany, is 2:1 and 7:1, respectively. The corresponding mean values in Europe are 1 ± 1:1 and 4 ± 2:1, and in Switzerland 1.5:1 and 6:1 (Fig. 2 A and B) [15]. In the USA, there are four surgeons for every pathologist.

### Influence of different working time models on working life

**Fig. 3:**
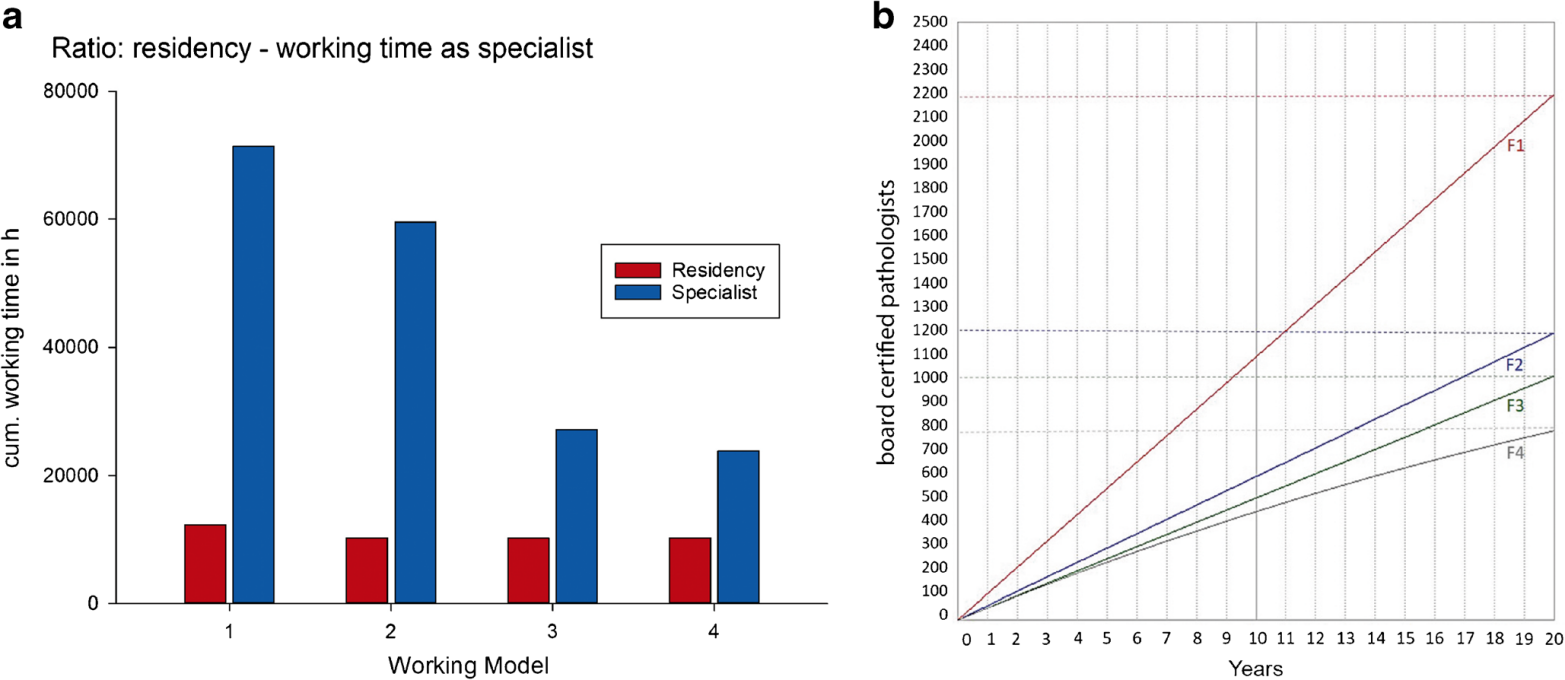
**A** Different working time models and their effect on working life. **M1** Start of continuing training at the age of 26 with specialist recognition after 6 years of continuing training and a subsequent specialist activity of 35 years in full-time without interruptions with a continuous daily working time of 10 h. **M2** like M1 but with 8 h working day. **M3** start of continuing training at the age of 26 with specialist recognition after 6 years of full-time continuing training, followed by a total of 3 years of work interruption (e.g. parental leave) and a subsequent specialist activity of 32 years in part-time without interruptions with a continuous daily working time of 4 h. **M4** Start of continuing training at the age of 28, after 4 years of interruptions totaling 3 years and part-time work (0.5VK), specialist recognition after 11 years. After that 28 years of part-time specialist work (0.5VK). **B** Development of recognition of specialists (corresponding to 1.0 UK) under different assumptions. **F1** Model with 115 new specialist qualifications per year and full-time job. After 20 years and 60 pathologists leaving the profession, the average European pathologist density of 2800 pathologists is reached. **F2** 60 new specialist qualifications per year and full-time job lead to the number being maintained. **F3** 60 new specialist recognitions per year and a 30% quota of specialists with 0.5VK part-time work. **F4** 60 new specialist recognitions per year and a 30% quota of specialists with 0.5VK which increases continuously to 70% within 20 years

To illustrate the effect of part-time models, the ratio of time spent as a residency (R) to time spent as a board-certified pathologist (BCP) was calculated under four different assumptions and is shown in Fig. 3A. Starting from a fixed R of 6 years or 10,200 h, a classic career with an assumed 50-h working week without work interruptions (model M1) and a modern model with adherence to the standard working time of 40 h, work interruptions and part-time work (model M4) represent both ends of the given spectrum. In model M1, the cumulative lifetime working time is around 84,000 h with a ratio of R to BCP of 1:6. In model M4, this is compared with 34,000-h lifetime working time with a R_BCP ratio of 1:2.3. In the two intermediate models, lifetime working times of 69,700 and 47,400 h are achieved, respectively. Figure 3 B shows a model of the development of board-certified pathologists over the next 20 years. Assuming a continuous increase in part-time work to 50% 0.5 full-time (FT) employment relationships, this would result in a loss of about 400 1.0 FT equivalents in 20 years. Assuming representativity of our survey (see next paragraph) and in the absence of compensation, dispensing with part-time employees would result in an 18% reduction of a board-certified pathologist (about 10% FT equivalent).

### Survey among heads of university institutes

**Fig. 4:**
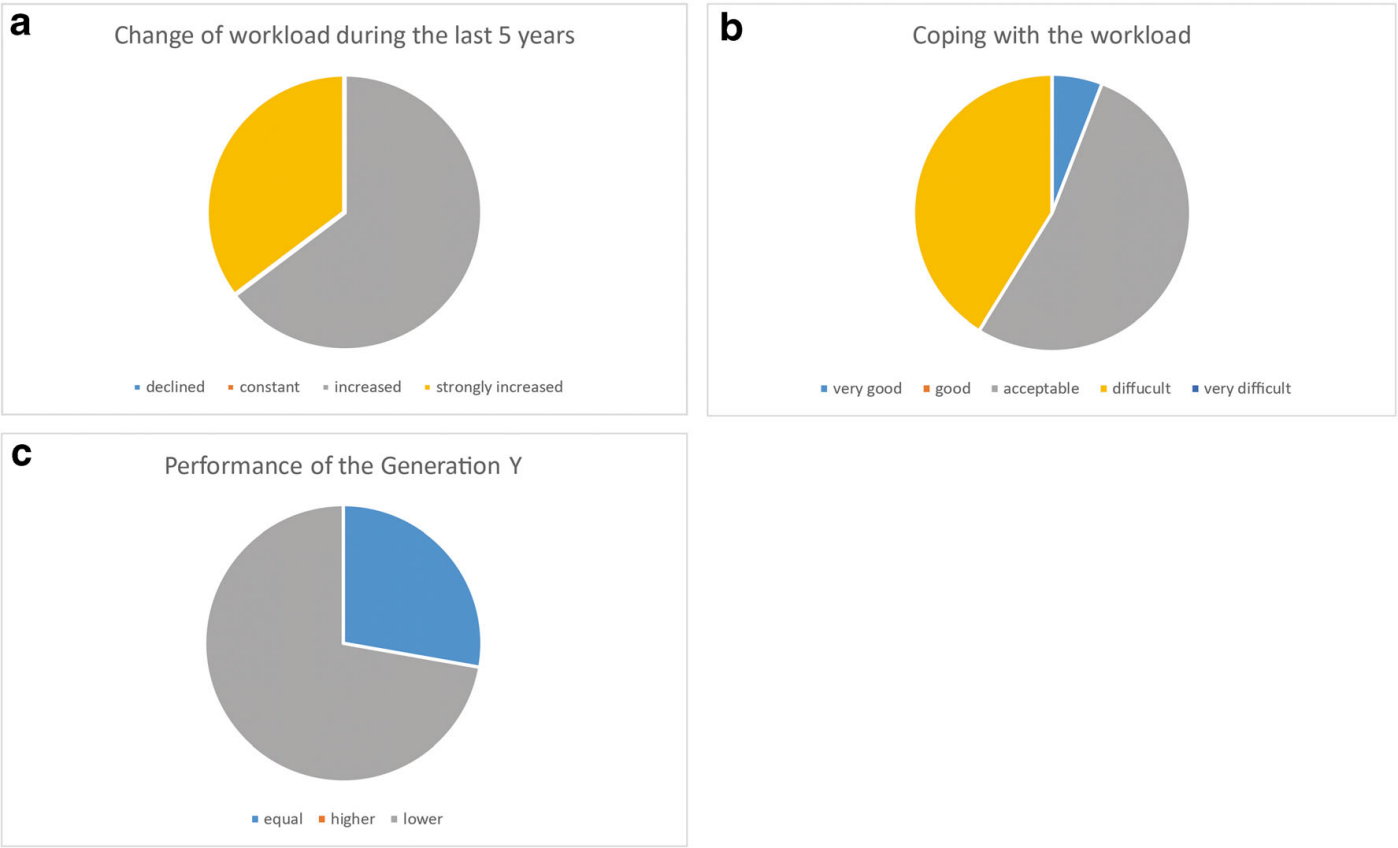
Survey results from 18 university pathology institutes. The workload increase over the last 5 years (**A**) and can mostly be copied only with difficulties (**B**). The estimation of the dedication of the Generation Y is discrepant with 28% seeing no differences in comparison to the baby boomer while 72% see a lower willingness to perform (**C**)

In an anonymous online survey, 37 university institutes in Germany were contacted. Eighteen of these institutes responded with a response rate of 49.6% (Table 1). With the exception of autopsies, the survey shows an increase in the areas of histology and interdisciplinary case conferences. On average, the institutes train seven residents simultaneously. The numerical ratio between medical specialists and continuing education assistants is almost 1:1. Only one institute rated the handling of the workload as very good, while the others were barely able to cope with the workload or did so with difficulty. There are different answers to the question of how the performance of the generation of 1980–1999 (Generation Y) can be assessed in comparison with the baby boomers. About a quarter of the responders see no differences, while 72% rate the performance or willingness to perform of the younger generation less highly (Fig. 4). Those who saw a lower willingness to perform among the younger generation estimated it at 68% (range: 30–80%; baby boomers = 100%).

**Table 1:** Survey results among 18 university pathology institutes

Question	Results
Case development histology in the 10 years	Average: + 26% (range: − 15%; + 110%)
Case development autopsy in the 10 years	Medium: − 34% (range: − 70%; + 50%)
Staff requirements for interdisciplinary conferences	Average: 1.5 (range: 0.3%; 3%)
Change in the need for conferences in 10 years	Average: 232% (range: 130%; 500%)
Number of training assistants (persons):	Medium: 7 (range: 2; 14)
Number of part-time working residents:	Average: 0.7 (range: 0; 3)
Number of medical specialists (persons):	Medium: 7 (range: 2; 13)
Number of part-time working specialists:	Average: 1.3 (range: 0; 3)

## Discussion

Recently, there have been an increasing number of reports about shortages of doctors in various areas of health care in different countries [4–12, 19–21]. The aim of this study is to examine the situation of pathology in Germany. Pathology is one of the smaller disciplines in terms of the number of active specialists, with about 1700 colleagues (as of 2017). Independent of this, however, it is an important cross-sectional discipline, whose efficiency is of system-critical relevance for numerous other disciplines. In comparison to other European countries as well as Canada and the USA, the situation in Germany is paradoxical. In relation to the population, the general physician density in Germany is particularly high, while the number of pathologists is the second-lowest among the countries examined here. At this point, it must be underlined that the comparability between the different countries is hampered. Autopsy for example is not the duty of pathology in all countries. On the other hand, in some countries, especially the English-speaking countries, the subject of pathology also includes microbiology and/or clinical chemistry. There are also considerable differing screening programs that influence the workload of pathologists. Moreover, there are also considerable differences between countries regarding the other medical specialties that are set into relation to the numbers of pathologists. These facts have to be stated as a clear limitation of this study. Nevertheless, in our opinion, it allows appropriate conclusions, as the results show a clear trend.

If the German system has a particularly high workload for pathologists, the legitimate question arises how this workload can be managed in the future. In order to maintain a constant number of about 1700 pathologists in Germany, an average number of 50 and 60 board certifications must be guaranteed per year. However, this presupposes that the rate of part-time employees among junior specialists is no higher than the number of retired specialists. It is extremely unlikely that this will be the case in the future. In the 2018 survey of the National Association of Statutory Health Insurance Physicians, the compatibility of family and career as well as regulated and flexible working hours were stated as important or very important expectations by 81.4% to 94.6% of the participating medical students. In contrast, subject- and career-related expectations (broad spectrum, research, career) are of less relevance, which were considered important or very important by 36% and 69.4%, respectively [22]. The desire for part-time work in medicine is also evident from the results of the KarMed study. Sixteen percent of the physicians and 50% of the female physicians in the 4th year of continuing education wanted to work part-time. As soon as children are involved, this proportion rises to 82% for female doctors, while the proportion remains the same for male doctors [23]. Almost 60% of female doctors plan to work part-time either for their entire career or after a few years of further training [24, 25]. It can, therefore, be assumed that the proportion of part-time employees in medicine will continue to increase in the future.

Among the models shown in Fig. 3A, the M3 or M4 model is much more realistic than the M1 model. On the other hand, part-time working enables a relevant number of physicians to qualify and stay in their profession. It is not only in the author’s opinion that part-time working is a major achievement of our modern societies. A further trend that complicates the situation is the permanently increasing workload that is documented by the results of our survey under university institutes in Germany. This is in concordance with the results reported by Warth et al. who analyzed the effects of personalized medicine and demographic change on the workload for the pathology [26]. A survey conducted by the Royal College of Pathologists in the United Kingdom showed even more serious results in this respect, with only 3% of the institutes having sufficient staff and high rates of replacement doctors needed in the institutes to cope with the workload for pathology. In addition, there was an unfavorable age structure of pathologists [13]. With regard to the performance/willingness of the next generation—so-called Generation Y—our survey revealed that almost three-quarters of the respondents felt that these colleagues performed less than the previous generation (Fig. 4). The new generation, on the other hand, is said to be highly self-confident and willing to perform. However, the latter is at the same time related to their own benefit. Prestige and income, on the other hand, tend to be of low significance [27–29]. It is therefore very likely that in Germany, a significantly higher number of pathologists will be needed to guarantee medical care. The necessity for such a development could be deduced from individual existing studies, which assume that the error frequency of pathologists correlates with the number of hours per week [30] and that pathologists are confronted with cut injuries and occupational diseases affecting the musculoskeletal system, the skin, the eyes but also the psyche at a relevant frequency. The frequency of burnout or depression is reported to be 12.3% and 16.7% [31, 32].

Measures to counteract an emerging bottleneck should include initiatives to increase the number of pathologists as well as the development of opportunities to rationalize the diagnostic and organizational effort in daily routine. It is beyond the scope of this article to provide ready-to-use solutions for these complex problems. However, it may be opportune to provide at least some suggestions for a future discussion. It is trivial that a significant increase of residents in training is an essential basic need to avoid part. For the recruitment of a sufficient number of young physicians, the visibility of the subject of pathology at the medical schools must be given. The additional efforts for the training of the residents could be reduced by offering an additional structured training program outside the institutes on a federal level which does not exist on this level. A way to heighten the speed of qualifying junior pathologist would be to reduce the duration of the training which is in Germany with 6 years among the longest in Europe. A financial compensation for the institutes that are engaged in the training of residents seems reasonable. The delegation of some of the pathologist’s tasks to especially qualified assistance staff could ease the situation. In the form of perfectly communicating systems, automation and digitalization could offer enormous potential for relieving pathologists in their daily work. A focus towards the essential diagnostic workup would be the result. While this article is focusing on the German situation, the key findings of this study have implications for many if not all European countries, northern America and probably at least some countries in Asia. While news of the lay press report about already existing problems in coping the changes in the daily routine of pathologist for example in United Kingdom or Italy, this is currently not the case in Germany. However, it has to be recognized that the already existing problems are compensated by the longer hour working pathologists. Reliable calculations are urgently needed to estimate the workforces in each country to monitor and if necessary adapt the number of residents to meet the demands on specialists.

## Conclusion

The present study shows that the current workload in German pathology institutes is handled by an extraordinarily small number of pathologists in international comparison. An increasing number of part-time physicians will lead to a further concentration of work and thus might aggravate an already difficult situation. It can also be assumed that future generations will no longer be willing to carry the increasingly heavy workload. It therefore seems urgently necessary to develop concepts that can counteract this expected bottleneck. The German professional organizations and societies already faced the emerging problems and discussing the situation and possible solution with their members during round table meetings. Here, a training offensive, a reform of the training as well as a forced use of modern digital technologies could offer solutions.

## Data Availability

Data except the results of the survey can be requested from the corresponding author.
